# Characterization of a Near-Complete Genome Sequence of a Chaphamaparvovirus from an Australian Boobook Owl (Ninox boobook)

**DOI:** 10.1128/mra.00249-22

**Published:** 2022-04-19

**Authors:** Subir Sarker, Ajani Athukorala, David N. Phalen

**Affiliations:** a Department of Microbiology, Anatomy, Physiology and Pharmacology, School of Agriculture, Biomedicine and Environment, La Trobe University, Melbourne, Victoria, Australia; b Sydney School of Veterinary Science, Faculty of Science, The University of Sydney, Sydney, New South Wales, Australia; c Schubot Exotic Bird Health, Texas A&M College of Veterinary Medicine and Biomedical Sciences, College Station, Texas, USA; Queens College CUNY

## Abstract

This study reports a complete genome sequence of a variant of psittacine chaphamaparvovirus 2 detected in kidney tissue from an Australian boobook (Ninox boobook), compiled using next-generation sequencing. The genome was 4,312 bp long, encoding four open reading frames. The detection of this variant in boobook represents a significant host-switching event.

## ANNOUNCEMENT

Chaphamaparvoviruses (ChPVs) are members of the family *Parvoviridae* and subfamily *Hamaparvovirinae*; they are nonenveloped, icosahedral viruses with a linear single-stranded DNA genome sequence of ~4.0 to 4.5 kb (1). They encode two genes: a nonstructural replicase gene (NS) and a capsid (VP) gene (2, 3). ChPVs are likely to be widespread in nature and have been detected in the feces of birds (4–6) and mammals (7); a ChPV causes renal disease in laboratory mice (8). Recently, two novel ChPVs (psittacine chaphamaparvovirus 1 and 2 [PsChPV-1 and PsChPV-2, respectively]) was detected in the liver of rainbow lorikeets (Trichoglossus moluccanus) (9) and fecal materials of *Neophema* birds (10) in Australia. Here, we report a near-complete genome sequence of a variant of PsChPV-2 (PsChPV-2v.1) detected in the kidney of an Australian boobook (Ninox boobook).

Kidney tissue was collected from a dead boobook submitted to the Sydney School of Veterinary Science (34°0′10.61″S, 150°37′27.84″E), between December 2018 and April 2019. DNA was extracted using the PureLink genomic DNA minikit (Invitrogen, CA, USA). The library was prepared using an Illumina DNA prep kit (San Diego, CA, USA), starting with 250 ng DNA (6). The quality and quantity of the prepared library were assessed by the Australian Genome Research Facility (AGRF), Melbourne, Australia, and the library was sequenced using the Illumina NovaSeq sequencing platform, generating 150-bp paired-end reads.

The sequencing data were analyzed as per an established pipeline (11–14) using Geneious version 10.2.2 (Biomatters, New Zealand) and CLC Genomics Workbench version 9.5.4. Briefly, 37,770,262 raw reads were preprocessed to remove Illumina adapters, ambiguous base calls, and poor-quality reads (reads trimmed using a quality score limit of 0.05; ambiguous nucleotides up to 15 bp trimmed using CLC Genomics Workbench), followed by mapping against barn owl (Tyto alba) (15) and Escherichia coli (GenBank accession no. U00096) sequences to remove nonviral DNA. A total of 37,612,162 trimmed and unmapped reads were used as input data for the *de novo* default assembler in CLC Genomics Workbench version 9.5.4. This resulted in the generation of a linear 4,312-bp contig, identified as a PsChPV-2 genome, with an average coverage of 70.26×. Annotation of the assembled genome was performed using Geneious version 10.2.2. All software was used with default parameters except where stated.

The genome is 4,312 bp long, with a G+C content of 41.4%. Comparative analysis of the predicted open reading frames (ORFs) of PsChPV-2v.1 was conducted using BLASTX and BLASTP; the ORFs encoding the NS1 and VP1 proteins share 100% and 99.8% identity, respectively, with the corresponding proteins of previously isolated PsChPV-2 (GenBank accession no. MZ364297) (10). As in other parvoviruses, the complete NS1 gene of PsChPV-2v.1 was 671 amino acids (aa) long and encodes the helicase, including the conserved ATP- or GTP-binding Walker A loop (GPxNTGKT/S; _317_**GP**S**NTGKS**_324_), Walker B (xxxWEE; _355_IGV**WEE**_360_), Walker B′ (KQxxEGxxxxxPxK; _372_**KQ**VL**EG**MQTSI**P**I**K**_385_), and Walker C (PxxxTxN; _396_**P**III**T**S**N**_402_) amino acid motifs (conserved amino acids are indicated in bold). In addition, the NS1 protein contains two conserved replication initiator (endonuclease) motifs, xxHuHxxxx (IF_108_**H**V**H**_110_AMLQ) and YxxK (_166_**Y**LM**K**_169_) (“u” indicates a hydrophobic residue).

This study highlights evidence of PsChPV-2 infection in kidney tissue from an Australian boobook. This expands the host range of PsChPV-2 to include an entirely unrelated species and suggests that at least some ChPVs may have a wide host range.

### Data availability.

The complete parvovirus genome sequence from *N. boobook* and the data that support the findings of this study have been deposited at DDBJ/ENA/GenBank under the accession no. OL762454. The version described in this paper is the first version, OL762454.1. The raw sequencing data from this study have been deposited at the NCBI Sequence Read Archive (SRA) under the accession no. SRR17163735 (BioProject accession no. PRJNA787018).
